# A Study on the Influencing Factors of Continued Intention to Use MOOCs: UTAUT Model and CCC Moderating Effect

**DOI:** 10.3389/fpsyg.2021.528259

**Published:** 2021-08-04

**Authors:** Yalin Li, Min Zhao

**Affiliations:** ^1^Business School, Suqian University, Suqian, China; ^2^School of Information and Communication Engineering, Hubei University of Economics, Wuhan, China

**Keywords:** UTAUT, MOOCs, connected classroom climate, continued intention to use MOOCs, moderating effect

## Abstract

Massive Open Online Courses (MOOCs) is the product of “internet + education,” which offer the open educational resources to global students. This study analyzed the factors influencing the continued intention to use the MOOCs by students. To achieve research objectives, this study integrated the unified theory of acceptance and use of technology (UTAUT) and connected classroom climate (CCC). In this study, 312 valid samples were used to verify the hypothesis proposed with the help of structural equation modeling and PROCESS. The results showed that the factors of UTAUT model (performance expectancy, effort expectancy, social influence, and facilitating conditions) had significant positive effects on continued intention to use MOOCs. More importantly, there was a significant moderating effect of CCC between UTAUT and the continued intention to use MOOCs. Based on this research the findings, implications and limitations are discussed.

## Introduction

A Massive Open Online Course (MOOC) is usually a free online course offered to anyone who wants to sign up to study. The registered students may download the video and content of courses, fulfill the assignments, and conduct quizzes with the help of MOOCs platform (Fianu et al., 2018), which was first proposed in 2008 by Dave and Bryan (Bederson et al., 2015) and first used the term MOOC by Downes and Siemens in 2008 (García-Peñalvo et al., 2018; Fang et al., 2019). MOOC provides students with new learning opportunities and new professional knowledge and skills through online courses (Barak et al., 2016; Evans et al., 2016; Watted and Barak, 2018). MOOCs are regarded as a revolution in the field of education and more people can get quality education resources through MOOCs (Evans et al., 2016; Wu and Chen, 2017). And most of the students are to learn a new concept or improve their knowledge by enrolling in MOOCs (Arpaci et al., 2020). With their advantages of without space and time constraints (Porter et al., 2014), many universities around the world put the development MOOC on the agenda (Sharrock, 2015). As of the end of 2018, more than 900 universities had created 11,400 MOOCs involving over 101 million learners around the world (Sun et al., 2020).

Although MOOCs were held up as an educational innovation (Joo et al., 2018) and had experienced rapid development in recent years (Konstan et al., 2015), there were still some criticisms of MOOCs (Cagiltay et al., 2020). For instance, MOOCs are facing a serious problem of low completion rates (Macleod et al., 2015; Li et al., 2018) or high dropout rates (Cagiltay et al., 2020). According to some estimates, only 15% of registrants were able to complete the course (Arpaci et al., 2020). What was more serious was that the low completion rates of MOOCs had never been improved over 6 years (Sun et al., 2020).

Generally speaking, the initial participation of learners is the first step in the successful implementation of the MOOC program and the key motivation for the ultimate success is the continuous participation and use (Wan et al., 2020). Although many students are attracted by the new teaching model and perfect functions of MOOCs and decide to register and acquire relevant knowledge, they somehow drop out or give up studying due to personal or environmental factors, which results in a low completion rate or high dropout. Thus, it is very critical to study the factors that affect the acceptance and continued intention to use MOOCs (Briz-Ponce et al., 2017; Nikou and Economides, 2017; Chao, 2019).

A main reason of high rate dropout is a lack of face-to-face engagement in MOOCs, which may lead to the students being isolated and disconnected from others (Waugh and Su-Searle, 2014). The connected classroom climate (CCC), which is defined as “student-to-student perceptions of a supportive and cooperative communication environment in the classroom” (Dwyer et al., 2004) is very important. The online courses, such as the MOOCs are a computer-mediated environment, which the students considered to be negatively affected and creates communication challenges (Yang et al., 2019). Furthermore, the nature of an online course (e.g., MOOC) is the lack of face-to-face interaction between students and teachers, and interaction in the learning experience plays a more important role (MacLeod et al., 2017; Yang et al., 2019). It is very important to understand the influence of CCC on MOOCs learning in the computer-mediated environment, but there are only a few studies in this field.

The success of online courses, such as MOOCs, depends on the behavioral intention and use behavior to the new technology (Clay et al., 2009). In the past decades, scholars have done a lot of research on the usage intentions of new technology (Davis, 1989; Chen and Hwang, 2019). Among these theoretical achievements, the unified theory of acceptance and use of technology (UTAUT; Venkatesh et al., 2003) has been regarded as a more complete theory than TAM and other previous models for its greater predictive capacity (Okumus et al., 2016) and higher explanatory power (Barrane et al., 2018). And the UTAUT has been widely used to investigate the factors influencing individual usage intentions of new technology in different environments (Khechine et al., 2016). UTAUT is considered as the most and even the best predictive model (Alawadhi and Morris, 2008; Al-Shafi and Weerakkody, 2010). To precisely identify factors and the mechanism that affected University student's continued intention to use MOOCs, this study integrated the theory of UTAUT and CCC to build an optimized conceptual model.

## Literature Review

### Unified Theory of Acceptance and Use of Technology

Over the years, researchers have used theories of human behavior to research technology acceptance and usage intention (Rahman et al., 2017). Literature research showed that there were many theories to analyze technology acceptance, such as Theory of Reasoned Action (TRA), Theory of Planned Behavior (TPB), Social Cognitive Theory (SCT), Technology Acceptance Model (TAM), Extended Technology Acceptance Model (TAM2), Motivational Model (MM), Model of PC Utilization (MPCU), Innovation Diffusion Theory (IDT), and Unified Theory of Acceptance and Use of Technology (UTAUT) (Barrane et al., 2018; Fianu et al., 2018). Among these theories, UTAUT integrated the other eight theories to examine the behavioral intention of learners (Venkatesh et al., 2003), which contained four core variables: performance expectancy (PE), effort expectancy (EE), social influence (SI), and facilitating conditions (FC) and four moderation variables: gender, age, experience, and voluntariness of use (Venkatesh et al., 2003).

In the field of technology acceptance and usage intention, UTAUT is the most widely used theory at present (Barrane et al., 2018). A citation of research made in 2014 published 743 scientific papers in journals applying UTAUT model from 2003 to 2013 (Khechine et al., 2016). The UTAUT model is the most predictive model for technology acceptance (Alawadhi and Morris, 2008; Al-Shafi and Weerakkody, 2010) and it is the most powerful theories to explain IT acceptance and use (Al-Qeisi et al., 2015). The UTAUT model could explain up to 70% of the variance of usage behavior (Venkatesh et al., 2003) and was better than the eight individual models with an adjusted *R*^2^ of 69% (Venkatesh et al., 2003). Moreover the literature also indicates that the UTAUT is a reliable theoretical model to clarify the influencing factors of technology acceptance across the globe (Arif et al., 2018). Therefore, this study uses UTAUT model as the theoretical basis of the study.

### Connected Classroom Climate

Connected classroom climate means the degree of supportive and cooperative communication among students in classrooms (Dwyer et al., 2004). Research shows that the positive classroom climate is good for students (MacLeod et al., 2017) and it has a positive effect on the participation of students in the classroom teaching (Sidelinger and Booth-Butterfield, 2010). For example, positive classroom climates support students to participate in classroom activities, help students to meet their psychological needs and cultivate self-determined motivation (Carini et al., 2006; Joe et al., 2017). Moreover the research indicated that CCC has a positive influence on emotional learning (Johnson, 2009; Frisby and Martin, 2010), cognitive learning (Frisby and Martin, 2010), and self-regulation learning (Sidelinger and Booth-Butterfield, 2010) of students. These contribute to classroom learning of students (Dwyer et al., 2004).

The insufficiency in interaction between learners and teachers is the main reason for the low completion rate of MOOCs (Wang et al., 2017), which causes learners to feel isolated (Wang et al., 2017). With the help of suitable conditions, face-to-face learning groups can help learners improve their knowledge and skills, form group consciousness, influence learning motivation and results of students, and reduce dropout rates (Holliday and Said, 2008; Arendale and Hane, 2014). MOOCs depend on the computer-mediated environments and these technical factors affect student participation and create additional communication challenges (Rovai and Jordan, 2004). CCC is not only important for face-to-face classroom learning, but also for learning through online courses (MacLeod et al., 2017). However, few studies have been conducted on the influence of CCC on MOOCs learning, although some studies showed that the online environment will have an impact on CCC, which in turn will affect the impact of CCC on students (Ritter et al., 2010; Yang et al., 2019). In particular, the influence of CCC on MOOCs acceptance and continued intention to use has not been studied.

### Dropout in MOOCs

Although many students use the MOOCs platform for online course registration and learning, the dropout rate is very high (Zhang et al., 2018). It is estimated that the dropout rate of MOOCs is close to 90% on average (Xing et al., 2016), which has become one of the serious problems in learning through the MOOCs (Schuwer et al., 2015; Li et al., 2018; Ortega-Arranz et al., 2019).

Many scholars have studied the reasons for the high dropout rate of MOOCs (Xing, 2018). Research from the perspective of students is one of the main directions and a large number of studies have been carried out around this angle (Veletsianos and Shepherdson, 2016). For example, some studies have discussed the influence of factors, such as motivation levels, attitudes, and patterns of participation behavior of students on the high dropout rate (Xing, 2018). Furthermore, motivation is one of the strongest predictors of participation and efficiency for students in MOOCs (Barba et al., 2016). And the motivation factors are mainly related to the expected benefits for students, including career benefits, personal benefits, and educational benefits (Watted and Barak, 2018). The subjective norms and usefulness of the MOOCs as perceived by students affect their continued intention to use (Xu, 2015).

In addition, some scholars have studied the influencing factors of MOOCs learning from the perspective of teachers. For example, Khalil and Ebner (2013) found the role of interaction in the learning process of MOOCs and how the perceptions of interaction affected MOOC learning behavior and learning effect in students. Stephens-Martinez et al. (2014) pointed out that a MOOC instructor's views about different sources of information might affect the behavior and performance level of students in the learning process of MOOCs. Moreover, Watson et al. (2016) showed that the MOOC instructors could influence the MOOC learning results and attitudes of students by using social presence, teaching presence, and dissonance factors. Different instructional approaches in MOOCs also affect the final learning outcomes of students (García-Martín and García-Sánchez, 2020).

Furthermore, some scholars have analyzed the reasons for the high dropout rate of MOOCs from strategic and environmental perspectives. For example, Li et al. (2018) indicated that it was related to network benefit, user preference, and motivation to achieve for students to insist on completing MOOCs learning tasks. Alraimi et al. (2015) found that the MOOCs perceived reputation, perceived openness, perceived usefulness, and overall user satisfaction significantly influenced on the continuous use intention of MOOCs. Zhang et al. (2018) found that online forum and the interaction with teachers played an important role in motivating students' continuous use of MOOCs learning. Ortega-Arranz et al. (2019) provided empirical evidence to prove that the use of reward-based gamification strategies was one approach to promote student engagement and prevent dropout. In addition, patterns of MOOC features (Xing, 2018), computer self-efficacy, performance expectancy, and system quality (Fianu et al., 2018) and network externalities (Li et al., 2018) influenced MOOCs usage intention and dropout rates.

### Summary

One of the most important characteristics of MOOCs is flexibility of self-arrangement of learning schedule by making use of the contents and resources of MOOCs (Bruff et al., 2013). Since most MOOCs support autonomous learning and in essence, it can realize sub-synchronous learning, which makes MOOCs learners often fail to receive direct feedback from teachers and have certain barriers to interaction with others (Kop et al., 2011). In contrast with traditional teaching methods, this kind of teaching method of MOOCs makes students lack the sense of participation and cannot realize the real-time interaction as seen in a physical classroom (Chang et al., 2018). Research shows that students lack motivation to use MOOCs for short of interaction and feedback with teachers, lack of group interaction, and poor communication (Hone and El Said, 2016), which is one of the main reasons for the serious dropout problem of MOOCs. Only using UTAUT model to explain acceptance of MOOCs by students may not achieve an effective result, when considering the social context of MOOCs. To account for the social characteristics of MOOCs and analyze the acceptance of MOOCs, on the basis of integrating CCC theory, this study proposes a new UTAUT model to better explain the influencing factors of students continued intention to use MOOCs. We tried to address two research questions:

**Research Question 1**: What are the crucial factors affecting students continued intention to use MOOCs?

**Research Question 2**: What are the moderating effects of CCC between UTAUT and students continued intention to use MOOCs?

## Research Model and Hypotheses

UTAUT is a powerful theoretical method, which is widely used to support the measurement of technology acceptance in related education environments (Yang et al., 2019) and it is the basic theoretical framework of this study. At the same time, considering the influence of interaction on learning, this study extends the UTAUT model combined with CCC theory. In the UTAUT model, students' acceptance and use of technology are affected by performance expectancy (PE), social influence (SI), performance expectancy (PE), effort expectancy (EE), and facilitating conditions (FC) (Venkatesh et al., 2003). In addition to these four independent variables (PE, EE, SI, and FC), the original model of UTAUT also included four moderators (gender, age, voluntariness, and experience) for better explaining the adaptability of models in different organizations and different backgrounds (Chen and Hwang, 2019). However research showed that the moderating role of gender and age did not exist in online learning systems for college students (Marchewka et al., 2007). The samples in this study were all college students of similar age and background. Due to the high consistency of samples in the study, the four moderators in the UTAUT model were not taken into account. Therefore in this study, we propose that the four UTAUT variables (PE, EE, SI, and FC) will influence students' continued intention to use MOOCs.

**Figure 1:**
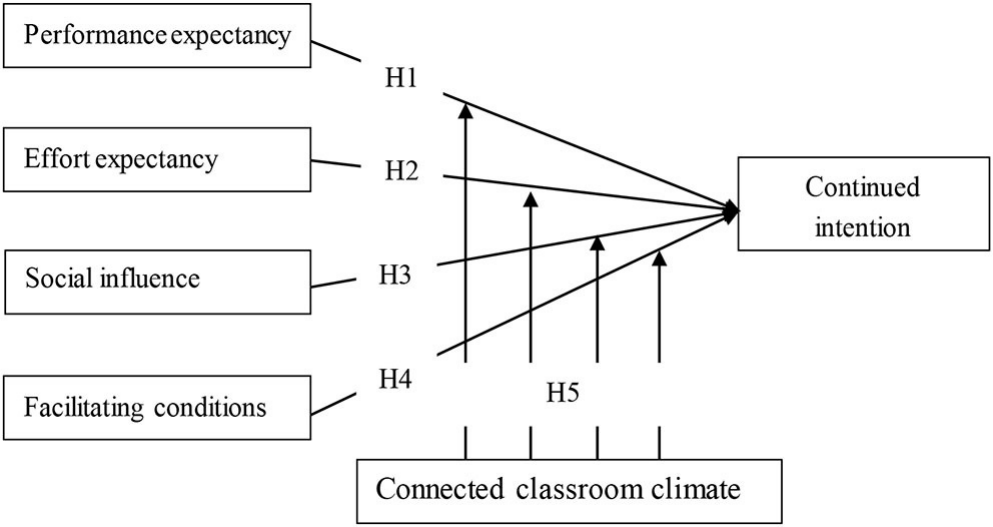
Theoretical model and hypotheses.

The research showed that social interaction between students and teachers and collaborative interaction among students were important to improve learning and actively participate in online discussions (Jung et al., 2002). Many studies have used CCC to examine the impact of the relationship between teachers and students and classroom atmosphere on learning in face-to-face environments (MacLeod et al., 2017), which is the representation of social interaction in the classroom. Lack of social interaction will increase students' pressure (Demakis and McAdams, 1994), difficulty adjusting to school, dropout tendency, and negative academic performance (McGrath et al., 2000). CCC can provide more opportunities for social interaction. With the help of social interaction the information value, emotional value, and hedonic value increases (Zhang et al., 2017), which effect the MOOCs acceptance (MA). Therefore, as shown in Figure 1, on the basis of existing research, this study integrates CCC theory and UTAUT theory, and deeply analyzes moderation role of CCC between the factors of UTAUT and continued intention to use MOOCs. The research hypotheses of this study are as follows:

H1: Performance expectancy has a positive effect on the intention of students continued to use MOOCs.

H2: Effort expectancy has a positive effect on the intention of students continued to use MOOCs.

H3: Social influence has a positive effect on the intention of students continued to use MOOCs.

H4: Facilitating conditions has a positive effect on the intention of students continued to use MOOCs.

H5: Connected classroom climate has moderating effect on the influence of UTAUT on students' continued intention to use MOOCs.

## Methodology

### Participants

According to the needs of the research, this study selected students with MOOCs learning experience as the survey objects. A total of 320 questionnaires in a University in China were issued in this study. Eight questionnaires were excluded because of incomplete filling and convergence of answers. Finally, 312 valid questionnaires were included in the analysis. The basic information of participants including gender, age, and grade were presented as demographic characteristics (see Table 1), in which female participants accounted for 55.10%, male participants accounted for 44.90%; 34.90% were under 20 years of age, 46.20% were between 21 and 23 years of age, and 18.90% were over 23 years of age. All the participants had the experience of using MOOCs.

**Table 1:** Demographics of the participants.

**Characteristics of the participants**	**Percentage**
**Gender:**	
Male	44.90
Female	55.10
**Age (years):**	
Under 20	34.90
21–23	46.20
Above 23	18.90
**Grade:**	
Freshman	17.30
Sophomore	34.30
Junior	28.20
Senior	20.20

### Measures

The survey used in this study and the research model contained six constructs with 39 items (see Table 2). In order to improve the content validity, the items of measurement are all from literature (Straub et al., 2004) and items were rephrased to fit the context of MOOCs. All items used in the present study were translated into Chinese using standard back-translation procedures (Brislin, 1970) with a seven-point Likert scale (“1 = strongly disagree” and “7 = strongly agree”). Moreover, to ensure the quality of the measurement scale the questionnaire were also reviewed by other professionals. These scales were described as follows.

**Table 2:** Measurement items.

**Constructs**	**Code**	**Items**	**References**
Performance expectancy	PE1	I believe that using MOOCs during my learning would be very useful	Venkatesh et al., 2003
	PE2	Using the MOOCs enables me to accomplish tasks more quickly	
	PE3	Using the MOOCs increases my productivity	
	PE4	If I use the MOOCs, I will increase my chances of getting a raise	
Effort expectancy	EE1	It would be easy for me to become skillful at using the MOOCs	
	EE2	My interaction with the MOOCs would be clear and understandable	
	EE3	I would find the MOOCs easy to use	
	EE4	Learning to operate the MOOCs is easy for me	
Social influence	SI1	People who influence my behavior think that I should use the MOOCs	
	SI2	People who are important to me think that I should use the MOOCs	
	SI3	The teacher of this course has been helpful in the use of the MOOCs	
	SI4	In general, the school has supported the use of the MOOCs	
Facilitating conditions	FC1	I have the resources necessary to use the MOOCs	
	FC2	I have the knowledge necessary to use the MOOCs	
	FC3	The MOOC is not compatible with other online courses I use	
	FC4	A specific person (or group) is available for assistance with MOOC difficulties	
Continued intention	CI1	I intend to continue to use MOOCs for assisting classroom learning	Lin and Wang, 2012; Wu and Chen, 2017
	CI2	I intend to continue to use MOOCs for enriching my knowledge	
	CI3	I will continue using MOOCs increasingly in the future	
	CI4	I will recommend other people to use MOOCs	
	CI5	Overall, I intend to continue to use MOOCs in the future	
Connected classroom climate	CCC1	I feel a sense of security in my class	Dwyer et al., 2004
	CCC2	I have common ground with my classmates	
	CCC3	I feel a strong bond with my classmates	
	CCC4	The students in my class share stories and experiences with one another	
	CCC5	The students in my class are friendly with one another	
	CCC6	The students in my class respect one another	
	CCC7	I feel included in class discussions in my class	
	CCC8	The students in my class are courteous with one another	
	CCC9	The students in my class praise one another	
	CCC10	The students in my class are concerned about one another	
	CCC11	The students in my class smile at one another	
	CCC12	The students in my class engage in small talk with one another	
	CCC13	The students in my class are non-judgmental with one another	
	CCC14	The students in my class laugh with one another	
	CCC15	The students in my class are supportive of one another	
	CCC16	The students in my class show interest in what one another is saying	
	CCC17	The students in my class cooperate with one another	
	CCC18	The students in my class feel comfortable with one another	

UTAUT scale consists of five dimensions, namely, performance expectancy (PE, four items, such as “I believe that using MOOCs during my learning would be very useful,” Chronbach's alphas = 0.902), effort expectancy (EE, four items, such as “It would be easy for me to become skillful at using the MOOCs,” Chronbach's alphas = 0.911), social influence (SI, four items, such as “People who influence my behavior think that I should use the MOOCs,” Chronbach's alphas = 0.860), facilitating conditions (FC, four items, such as “I have the resources necessary to use the MOOCs,” Chronbach's alphas = 0.838) and continued intention (CI, five items, such as “I intend to continue to use MOOCs for assisting classroom learning,” Chronbach's alphas = 0.924).

The CCC was measured by the scale of Dwyer et al. (2004), which included a single dimension with 18 items. One representative item of this scale is “I feel a sense of security in my class.” The overall reliability on the scale is 0.976.

All measurement scales reliability analysis show that the Cronbach's α coefficient exceeds the 0.70 threshold (Nunnally, 1978). The measure is reliable.

### Data Analysis Method

Data were collected voluntarily and anonymously *via* paper format and analyzed using SPSS 23.0 and AMOS 24.0. Using structural equation modeling (SEM) to test the relationship between independent variables and the dependent variable. The moderating effect of CCC was tested by the bootstrapping procedures and used the plug-in of PROCESS (Hayes, 2018), which integrates many of the functions of existing and popular published statistical tools for mediation and moderation analyses (Sun et al., 2014).

## Results

### Measurement Model Results

The purpose of this part is to test the reliability and validity of the research instruments used in this study. Specifically, the measurement model was assessed on four aspects: the item reliability, internal consistency reliability, convergent validity, and discriminant validity (Hair et al., 2011).

First, item reliability examines is estimated by evaluating the loadings of all the items on their latent variable. And the standardized loadings of the items should be 0.50 or higher, and ideally 0.70 or higher and all factor loadings should be statistically significant (Anderson and Gerbing, 1988). Also squared multiple correlation (SMC) is sometimes referred to as item reliability, which represents the extent to which a measured variance of a variable is explained by a latent factor and it should exceed 0.50 (Hair et al., 2010). Second, internal consistency was commonly measured by the composite reliability (CR). And the results of CR for each latent variable should be higher than 0.70. Third, convergent validity can be observed through Average Variance Extracted (AVE) testing. AVE for each latent variable should exceed 0.50 (Fornell and Larcker, 1981). Amos 24.0 is used to conduct confirmatory factor analysis (CFA, see Tables 3, 4) for an examination of the validity of the measure. All the standardized factor loadings exceed 0.70 (*p* < 0.001), AVE exceeds 0.50 (*p* < 0.001), CR exceeds 0.70 (*p* < 0.001), and SMC exceeds 0.50, indicating that the measure has adequate reliability and convergent validity.

**Table 3:** Reliability and convergent validity of UTAUT scale (*n* = 312).

**Construct**	**Indicator**	**Sig. test of parameters**				**Std**.	**Item reliability**	**Composite reliability**	**Convergence validity**	**Cronbach's α**
		**Unstd**.	**S.E**.	***t*-value**	***p***		**SMC**	**CR**	**AVE**	
Performance expectancy (*M* = 5.220, *SD* = 0.920)	PE1	1.000				0.850	0.723	06	0.706	0.902
	PE2	0.937	0.048	19.655	^***^	0.885	0.783			
	PE3	0.909	0.050	18.013	^***^	0.835	0.697			
	PE4	0.978	0.059	16.486	^***^	0.788	0.621			
Effort expectancy (*M* = 4.368, *SD* = 1.111)	EE1	1.000				0.872	0.760	0.911	0.719	0.911
	EE2	1.012	0.048	20.975	^***^	0.891	0.794			
	EE3	0.869	0.047	18.542	^***^	0.827	0.684			
	EE4	0.895	0.051	17.480	^***^	0.799	0.638			
Social influence (*M* = 4.946, *SD* = 0.968)	SI1	1.000				0.786	0.618	0.862	0.610	0.860
	SI2	1.085	0.077	14.013	^***^	0.786	0.618			
	SI3	1.073	0.073	14.631	^***^	0.821	0.674			
	SI4	1.030	0.080	12.920	^***^	0.729	0.531			
Facilitating conditions (*M* = 5.028, *SD* = 0.835)	FC1	1.000				0.737	0.543	0.842	0.571	0.838
	FC2	0.948	0.073	12.986	^***^	0.793	0.629			
	FC3	0.941	0.074	12.739	^***^	0.776	0.602			
	FC4	1.015	0.086	11.781	^***^	0.714	0.510			
Continued intention (*M* = 4.896, *SD* = 0.861)	CI1	1.000				0.811	0.658	0.928	0.721	0.924
	CI2	1.161	0.055	21.265	^***^	0.946	0.895			
	CI3	1.169	0.056	20.863	^***^	0.934	0.872			
	CI4	0.754	0.048	15.750	^***^	0.775	0.601			
	CI5	0.892	0.058	15.353	^***^	0.760	0.578			

*AVE, Average Variance Extracted; SMC, Square Multiple Correlation; CR, Composite Reliability*.

*** *p < 0.001*.

**Table 4:** Reliability and convergent validity of CCC (*n* = 312).

**Construct**	**Indicator**	**Sig. test of parameters**				**Std**.	**Item reliability**	**Composite reliability**	**Convergence validity**	**Cronbach's α**
		**Unstd**.	**S.E**.	***t*-value**	***p***		**SMC**	**CR**	**AVE**	
Connected classroom climate (*M* = 4.268, *SD* = 0.992)	CCC1	1.000				0.878	0.771	0.976	0.696	0.976
	CCC2	0.868	0.041	21.112	^***^	0.851	0.724			
	CCC3	0.911	0.044	20.856	^***^	0.846	0.716			
	CCC4	1.036	0.051	20.392	^***^	0.836	0.699			
	CCC5	1.032	0.052	19.748	^***^	0.822	0.676			
	CCC6	0.887	0.046	19.099	^***^	0.808	0.653			
	CCC7	0.946	0.045	21.163	^***^	0.852	0.726			
	CCC8	0.959	0.051	18.616	^***^	0.797	0.635			
	CCC9	0.938	0.042	22.231	^***^	0.872	0.760			
	CCC10	0.974	0.049	20.004	^***^	0.828	0.686			
	CCC11	0.897	0.042	21.107	^***^	0.851	0.724			
	CCC12	0.887	0.047	19.018	^***^	0.806	0.650			
	CCC13	1.036	0.050	20.710	^***^	0.843	0.711			
	CCC14	0.954	0.052	18.518	^***^	0.794	0.630			
	CCC15	0.922	0.049	18.829	^***^	0.802	0.643			
	CCC16	1.003	0.049	20.592	^***^	0.840	0.706			
	CCC17	0.880	0.045	19.474	^***^	0.816	0.666			
	CCC18	0.935	0.042	22.119	^***^	0.870	0.757			

*** *p < 0.001*.

*AVE, Average Variance Extracted; SMC, Square Multiple Correlation; CR, Composite Reliability*.

Discriminant validity reflects whether two factors are statistically different (Gan and Li, 2015). The discriminant validity of UTAUT scale evaluated by the means of AVE (Fornell and Larcker, 1981). As shown in Table 5, the square root of AVE for each variable (diagonal values in bold) was obviously larger than the respective correlation with other variables, which validates the discriminant validity of the constructs.

**Table 5:** Correlations and discriminant validity of the UTAUT scale.

**Construct**	**PE**	**EE**	**SI**	**FC**	**CI**	**CCC**
Performance Expectancy (PE)	**0.840**					
Effort Expectancy (EE)	0.345^**^	**0.848**				
Social influence (SI)	0.372^**^	0.270^**^	**0.781**			
Facilitating conditions (FC)	0.462^**^	0.441^**^	0.364^**^	**0.756**		
Continued intention (CI)	0.530^**^	0.464^**^	0.458^**^	0.568^**^	**0.849**	
Connected classroom climate(CCC)	0.189^**^	0.182^**^	0.240^**^	0.212^**^	0.365^**^	**0.834**

*1. Diagonal elements (in Bold) represent the square root of AVE for that construct*.

*2. Correlation is significant at the 0.01 level (2-tailed)*.

*3. AVE, Average Variance Extracted*.

*** *p < 0.001*.

As all data were self-reported by the sample, common method bias (CMB) may be found in the studies. Thus Harman's single factor test was conducted to assess whether the CMB existed (Podsakoff and Organ, 1986). According to the research of Podsakoff et al. (2003), if a factor accounts for most of the covariance of the variables, it provides evidence of the existence of CMB. The results from the exploratory factor analysis showed that the largest variance explained by an individual factor is 38.213% of the total variance and none of the factors could explain most of the variance, further indicating that CMB did not exist in this research (Vance et al., 2008).

To test for multicollinearity, the variance inflation factors (VIFs) were computed (Ramon et al., 2019). Results showed that the lowest value of VIFs was 1.091, and the highest value of VIFs was 1.499, where all the values of VIFs were below the conservative threshold of 5 (Hair et al., 2010). Therefore, multicollinearity was not serious in this study.

### Structural Model Results

**Figure 2:**
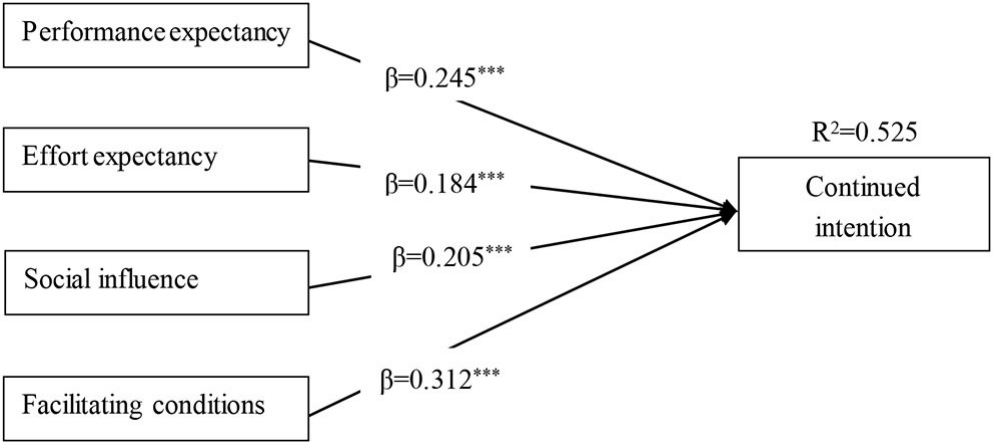
Results of the proposed SEM. ***p < 0.001.

The structural model is mainly analyzed in two aspects: testing the significance levels of the path coefficients and the explanatory power (*R*^2^) of the model. The proposed hypotheses (H1–H4) have been tested using SEM. The results of the SEM analysis of the structural model indicated (as shown in Table 6 and Figure 2) that hypotheses H1–H4 are supported by the empirical data. PE, EE, SI, and FC have significant effects on continued intention to use MOOCs (β = 0.245, *p* < 0.001; β = 0.184, *p* < 0.001; β = 0.205, *p* < 0.001; β = 0.312, *p* < 0.001).

**Table 6:** Structural equation modeling (SEM) results.

**H**	**Relationship**	**Path coefficient**	***t*-value**	***p*-value**	**Direction**	**Decision**
H1	Performance expectancy → Continued intention	0.245	4.203	^***^	Positive	Supported
H2	Effort expectancy → Continued intention	0.184	3.364	^***^	Positive	Supported
H3	Social influence → Continued intention	0.205	3.702	^***^	Positive	Supported
H4	Facilitating conditions → Continued intention	0.312	4.562	^***^	Positive	Supported

*** *p < 0.001*.

*χ^2^ = 370.681, d.f. = 179, χ^2^/df = 2.071(<3), GFI = 0.901(>0.90), NFI = 0.922(>0.90), CFI = 0.958(>0.90), RMSEA = 0.059(<0.08)*.

*GFI, Goodness-of-fit index; NFI, Normed fit index; CFI, Comparative fit index; RMSEA, Root mean square error of approximation*.

The explanatory power (*R*^2^) for the endogenous variable is regarded as the essential criterion for the structural model test (Henseler et al., 2009). At present, there is no unified agreed standard for the optimal value for *R*^2^. This study refers to the standards of Cohen (1988) on *R*^2^, who suggested that *R*^2^ values of 0.02, 0.13, and 0.26 represented small, medium, and large explanatory power, respectively. In the research model of this study, the *R*^2^ values for continued intention to use MOOCs was 0.525, which indicated that the model had generally substantial explanatory power. The above results confirm the hypotheses (H1–H4) proposed in this study.

### The Moderating Effects Analysis

To test moderation effect of CCC between PE, EE, SI, FC and continued intention to use MOOCs, a moderation analysis was conducted to test the hypothesis (H5) by the bootstrapping procedures and used Hayes (2018) statistical procedure PROCESS in SPSS.

The results of PROCESS are shown in Table 7. The interaction between CCC and PE, EE, SI, and FC was related to continued intention to use MOOCs (β = 0.195, 0.121, 0.113, and 0.266; *p* < 0.001, *p* < 0.001, *p* = 0.003, and *p* < 0.001, respectively), which means that the regression coefficient of independent variables (PE, EE, SI, and FC) to the dependent variable (CI) will increase by 0.195, 0.121, 0.113, and 0.266 units with the moderation variable of CCC increasing one unit. The moderation role of CCC exists. In addition we used the information in the results to calculate simple effects at low and high levels of CCC (see Table 8). The results show that compared with CCC at low level the influence of independent variable on the dependent variable is further enhanced under the high-level CCC. Hypothesis 5 of this study is confirmed.

**Table 7:** Moderation effect of CCC between UTAUT and CI.

**Variable**	**Dependent variable: continued intention**			
	**β**	**Se**	***t***	***p***
Performance expectancy	−1.218	0.152	−8.019	^***^
Connected classroom climate	−1.288	0.203	−6.357	^***^
Performance expectancy × Connected classroom climate	0.195	0.037	5.279	^***^
*R* ^2^			0.407	
*F*			70.452^***^	
Effort expectancy	0.807	0.135	5.960	^***^
Connected classroom climate	−0.794	0.151	−5.278	^***^
Effort expectancy × Connected classroom climate	0.121	0.032	3.749	^***^
*R* ^2^			0.327	
*F*			49.884^***^	
Social influence	0.780	0.150	5.195	^***^
Connected classroom climate	−0.801	0.194	−4.135	^***^
Social influence × Connected classroom climate	0.113	0.038	2.996	0.003
*R* ^2^			0.299	
*F*			43.783^***^	
Facilitating conditions	1.581	0.159	9.962	^***^
Connected classroom climate	−1.600	0.203	−7.785	^***^
Facilitating conditions × Connected classroom climate	0.266	0.039	6.896	^***^
*R* ^2^			0.467	
*F*			90.011^***^	

*** *p < 0.001*.

**Table 8:** Conditional effects of the focal predictor at high and low levels of the moderator CCC.

**Independent variable**	**Dependent variable: CI**					
	**Level**	**Effect**	**se**	***T***	**Boot 95% LLCI**	**Boot 95% ULCI**
PE	High	0.578	0.049	11.892	0.482	0.674
	Low	0.190	0.064	2.967	0.064	0.317
EE	High	0.411	0.044	9.263	0.324	0.499
	Low	0.172	0.054	3.208	0.067	0.278
SI	High	0.410	0.048	8.513	0.315	0.505
	Low	0.187	0.070	2.676	0.049	0.324
FC	High	0.709	0.051	13.894	0.609	0.809
	Low	0.181	0.067	2.710	0.050	0.313

*CI, confidence interval; LL, lower limit; UL, upper limit*.

## Discussion

This study attempted to explore the following two questions: the key factors affecting students continued intention to use MOOCs and the moderating effect of the CCC. With the help of UTAUT theory and CCC theory, this research investigates the effects of performance expectancy, effort expectancy, social influence, facilitating conditions on continued intention to use MOOCs and the moderation effect of CCC. The results show that our model has a good explanatory power in predicting the continued intention to use MOOCs. Data were collected using a survey instrument and 312 valid samples were recruited from a public University in China. The data were analyzed by SEM and Process 3.0. Moreover, this research shows that factors such as performance expectancy, effort expectancy, social influence, and facilitating conditions have a significant impact on continued intention to use MOOCs and the CCC has a significant moderating effect between UTAUT and students continued intention to use MOOCs.

### Summary of Findings

The MOOCs provide a lot of meaningful opportunities for educational stakeholders, especially students in higher education (Sabi et al., 2016), which provides more high-quality resources for these students to learn. The study was motivated by the fact that the MOOCs have been widely criticized for the low completion rate. This study discusses the high dropout rate of students from the perspective of technology acceptance, namely, this study incorporated UTAUT (Venkatesh et al., 2003) and CCC (Dwyer et al., 2004) to examine the continued intention to use MOOCs.

This research showed that performance expectancy, effort expectancy, social influence, and facilitating conditions have a significant impact on the continued intention to use MOOCs. Moreover, our findings also have confirmed the moderation role of CCC in the effect of UTAUT on the continued intention to use MOOCs. Previous studies have shown that performance expectancy, effort expectancy, and social influence had an influence on behavioral intention (Venkatesh et al., 2003). This study confirms the existing research conclusions from a new perspective which performance expectancy, effort expectancy, and social influence have a positive impact on students' continued intention to use online courses during the learning process. In addition, this research also provides support for studies conducted by Děcman (2015), Wang et al. (2009), which showed that performance expectancy had a positive influence on usage intention.

The previous research had confirmed that the CCC is beneficial for students to learn, integrate, and retain in face-to-face environments (MacLeod et al., 2017). The current research results showed that the CCC as a moderator had positively impacted the relationship between UTAUT and the continued intention to use MOOCs, which enhanced the influence of UTAUT on continued intention to use MOOCs. This research has examined the CCC in computer-mediated environments, which is consistent with Yang et al. (2019) who found that CCC had a significant impact on cloud classroom acceptance.

### Theoretical Implications

This research integrated UTAUT and CCC to explain the continued intention to use MOOCs, which provided a new model to interpret online course learning. The results from the analysis of the structural equation model indicated that continued intention to use MOOCs by students could be influenced by learning environmental expectancy, such as performance expectancy, effort expectancy, social influence, and facilitating conditions. That is to say when considering the intention of continued participation in MOOCs by students, we should consider the acceptance of information technology according to the learning environments.

In addition, the research verified the role of CCC in computer-mediated environments and combined with the UTAUT model, the study analyzed the relationships between performance expectancy, effort expectancy, social influence, facilitating conditions with continued intention to use MOOCs and CCC. Because a person's learning should not be separated from the social environment, but depends on the interaction with others (Schunk, 2012). The findings in the research showed that the same principle also was applicable for MOOCs. These results broaden the knowledge of CCC in computer-mediated environments, which as a new social variables are essential for understanding the continued intention to use MOOCs.

### Practical Implications

Our research found that performance expectancy, effort expectancy, social influence, and facilitating conditions in the UTAUT model had direct positive effects on the continued intention to use MOOCs, which provided the theoretical basis for how to improve the continued intention to use MOOCs. Specifically, for the technology attributes of performance expectancy and effort expectancy, which mean that students attach great importance to the practicability and ease of use of the technology in question. Therefore, the teachers should focus on improving the ease of use and practicability of MOOCs, such as selecting technology that is more suitable for students.

In addition, social factors had a positive impact on the continued intention to use MOOCs. The teachers may proactively manage social influence by organizing forums for sharing best practices. Strengthening interaction between student–student interaction or instructor–student interaction is helpful to improve continued intention to use MOOCs. For example, providing a platform that offers personalized feedback, creates real-life context, and encourages social interaction and more student reflection (Johnson and Aragon, 2003; Fianu et al., 2018). As the case with studies conducted by Děcman (2015) and Wang et al. (2009), facilitating conditions had a positive influence on continued intention to use MOOCs. Hence, the universities should consider providing adequate infrastructural facilities and resources, such as more free MOOC resources, simpler learning process, computer equipment, and smooth network links, to the students which will promote the use of MOOCs.

This study and previous literatures showed that he CCC was considered to be very important in both traditional face-to-face classroom teaching (Johnson and LaBelle, 2015) and computer-mediated environments (Yang et al., 2019). Most importantly, the online environment presents a lot of additional communication challenges (Rovai and Jordan, 2004), which is even more important for online learning lacking of interaction. This study investigates the moderation relationship between technological factors (performance expectancy, effort expectancy, social influence, and facilitating conditions) and continued intention to use MOOCs by students. Our results show that CCC can improve the continued intention to use MOOCs, which can help educational administrators and online education implementers to improve the efficiency of online learning by cultivating CCC.

### Limitations and Future Study

Although the current research is of great implications, it is not without limitations. First, for the convenience of sample collection the participants of this study were obtained from only one University, whether the conclusion of this study can be extended to a wider range is questionable. In future, more diversified samples and larger samples size can be investigated to enrich the research conclusions. Second, for the convenience of data collection, this study collected data through questionnaires conducted by self-reported. Although this study tried to standardize the data collection process, as self-reported survey relies heavily on human memory, errors could occur (Sudman and Bradburn, 1973). Therefore in the future, other data collection methods, such as experimental method, can be considered to further improve the conclusions of this study. Third, this study analyzed the continued intention to use MOOCs from the perspective of UTAUT model and focused on the influence of UTAUT factors. Previous studies have shown that many factors affected the UTAUT model. In the future, the research can combine the characteristics of MOOCs, analyze the factors that affect UTAUT under the background of MOOCs, and further explore the factors that affect continued intention to use MOOCs.

## Data Availability Statement

The raw data supporting the conclusions of this article will be made available by the authors, without undue reservation, to any qualified researcher.

## Ethics Statement

Ethical review and approval was not required for the study on human participants in accordance with the local legislation and institutional requirements. Written informed consent for participation was not required for this study in accordance with the national legislation and the institutional requirements.

## Author Contributions

YL design, data collection, and write the manuscript. MZ analyzed data. All authors contributed to the article and approved the submitted version.

## Conflict of Interest

The authors declare that the research was conducted in the absence of any commercial or financial relationships that could be construed as a potential conflict of interest.

## Publisher's Note

All claims expressed in this article are solely those of the authors and do not necessarily represent those of their affiliated organizations, or those of the publisher, the editors and the reviewers. Any product that may be evaluated in this article, or claim that may be made by its manufacturer, is not guaranteed or endorsed by the publisher.
